# The prognostic value of the lactate/albumin ratio for predicting mortality in septic patients presenting to the emergency department: a prospective study

**DOI:** 10.1080/07853890.2021.2009125

**Published:** 2021-12-02

**Authors:** Ralphe Bou Chebl, Mirabelle Geha, Mohamad Assaf, Nadim Kattouf, Saadeddine Haidar, Karim Abdeldaem, Nour Halawi, Mohamed Khamis, Maha Makki, Hani Tamim, Gilbert Abou Dagher

**Affiliations:** a Department of Emergency Medicine, American University of Beirut, Beirut, Lebanon; b Department of Internal Medicine, American University of Beirut, Beirut, Lebanon

**Keywords:** Outcome, mortality, infection, sepsis, prognosis, emergency medicine, lactate/albumin, lactate, albumin

## Abstract

**Objectives:**

Lactate/albumin (L/A) ratio is a biomarker in sepsis that has been shown to outperform lactate. This prospective study aims to validate the superior prognostic value of the L/A ratio to lactate in sepsis and septic shock.

**Methods:**

Prospective cohort conducted from September 2018 till February 2021 on adult patients presenting to the Emergency Department (ED) at a tertiary care centre with sepsis or septic shock. The primary outcome was the prognostic value of the L/A ratio compared to lactate with regards to mortality.

**Results:**

A total of 939 septic patients were included throughout the study period. A total of 236 patients developed septic shock. The AUC value of the L/A ratio in septic patients was 0.65 (95% CI 0.61–0.70) and was higher than that of lactate alone 0.60 (95% CI 0.55–0.64) with a *p* < .0001. The optimal L/A ratio cut-off threshold that separated survivors from non-survivors was found to be 0.115 for all septic patients. The AUC of the L/A ratio was significantly higher for patients with a lactate ≥2 mmol/L: 0.69 (95% CI 0.64–0.74) versus 0.60 (95% CI 0.54–0.66) with a *p* < .0001 as well as for patients with an albumin level less than 30 g/L (AUC = 0.69 95% CI= 0.62–0.75 vs AUC= 0.66 95% CI= 0.59–0.73, *p* = .04). Among septic shock patients there was no statically significant difference in the AUC value of the L/A ratio compared to lactate (0.53 95% CI 0.45–0.61 vs 0.50 95% CI 0.43–0.58 respectively with a *p*-value = .11).

**Conclusions:**

The L/A ratio is a better predictor of in-patient mortality than lactate in sepsis patients. This superiority was not found in the septic shock subgroup. Our results encourage the use of the ratio early in the ED as a superior prognostic tool in sepsis patients.

**Key messages**
1. We aimed to assess the prognostic usefulness of the Lactate/Albumin ratio compared to lactate alone in septic and septic shock patients.
2. The L/A ratio proved to be a better predictor of in-patient mortality than lactate alone in sepsis patients. This pattern also applies across various subgroups in our study (malignancy, diabetics, age above 65, lactate level less than 2 mmol/L, albumin less than 30 g/L). Our results favour the use of the L/A ratio over lactate alone in patients with sepsis and the previously mentioned subgroups.
3. Our results do not favour the use of the ratio instead of lactate in septic shock patients as there was no statistically significant difference between the AUCs of the ratio and lactate alone.

## Introduction

### Background

A serum “biomarker” is a readily measurable laboratory analyte. When appropriately interpreted in a clinical setting, it has diagnostic and prognostic values, and guides patient management and physician decision-making [1]. This is particularly important in sepsis and septic shock where early identification and antibiotic administration in busy Emergency Departments (EDs), can improve patient mortality [2]. Sepsis, despite advances in medical care, remains a major healthcare burden with significant morbidity and mortality, and remains one of the most common presentations to the ED [3]. Lactate is one of the most studied sepsis biomarkers in the literature and several studies have shown that elevated levels are associated with increased mortality [4]. Several factors can influence lactate levels which can limit its prognostic value in patients with sepsis and septic shock [5–7].

### Importance

Previous studies have investigated the lactate/albumin ratio (L/A) as a biomarker in sepsis and septic shock; however, they were either retrospective or small in sample size. The ratio was shown to outperform lactate as a prognostic tool in sepsis.

### Goals of this investigation

This study was prospective in nature and aimed to compare the prognostic value of L/A versus lactate in sepsis patients.

## Methods

### Design

This was a prospective cohort study of adult patients presenting to the ED with sepsis or septic shock. We aimed to evaluate the prognostic value of the L/A compared to lactate. Research assistants scanned the ED dashboard 24 h seven days a week. If a patient was suspected of having sepsis (flagged by the electronic medical record), the research assistant approached the family to obtain a written, voluntary and informed consent. This study was approved by the Institutional Review Board with a protocol number BIO-2018-0133.

### Study population and setting

This study was conducted at the ED of a tertiary care centre between September 2018 and February 2021. All patients diagnosed with sepsis or septic shock were included in the study. Sepsis was defined according to the sepsis-3 definition as a life-threatening organ dysfunction caused by a dysregulated host response to infection [8]. Organ dysfunction can be identified as an acute change in total SOFA (sequential organ failure assessment- which incorporates six variables: the respiratory status, coagulation status, liver function, cardiovascular status, central nervous system status and renal status) score ≥2 points consequent to the infection [8]. The baseline SOFA score can be assumed to be zero in patients not known to have pre-existing organ dysfunction. Septic shock was defined as having sepsis with any of the following: the need for vasopressors to keep the mean arterial pressure ≥ 65 mmHg or a lactate level > 2 (mmol/L) given the patient is not hypovolemic (remains hypovolemic clinically despite adequate volume resuscitation). Adequate volume resuscitation was left to the discretion of the treating physician as there is variability in the literature on this topic [8]. The exclusion criteria were age < 18 years, cardiac arrest on presentation, pregnancy, trauma patients, patients discharged from the emergency department, patients not meeting sepsis-3 criteria and patients who did not have a final diagnosis of sepsis (antibiotics stopped at 24 h).

### Interventions and measurements

We collected the following information from sepsis patients: vital signs upon presentation to the ED; co-morbidities, infection site, blood work (Complete Blood Count (CBC), Blood Urea Nitrogen (BUN), Creatinine, Electrolytes, Bilirubin, Lactate, Liver Enzymes, two blood cultures, urine analysis and urine cultures) in addition to a blood albumin level, use of vasopressors, antibiotics, steroids as well as patient disposition. Patients were followed throughout their hospital stay to determine length of hospital stay and in-hospital mortality. All variables were collected from patient charts that can be accessed through the Electronic Health Record system.

### Outcome measures

The primary outcome was the prognostic value of the L/A ratio (Albumin in g/L) compared to lactate (mmol/L) with regards to in-hospital mortality. The secondary outcomes were to determine the optimal cut-off of the L/A ratio that discriminates between survivors and non-survivors, and to examine the prognostic value of the ratio in subgroup populations (Lactate < 2; Lactate ≥ 2; septic shock; diabetes; malignancy; chronic kidney disease; age; source of infection; albumin < 30; albumin ≥30; septic shock and end-stage liver disease).

### Data analysis

In the univariate analysis, the distribution of the vital signs upon presentation to the ED, co-morbidities, laboratory analysis, blood and urine cultures, urine analysis, vasopressors, antibiotics and steroids use and patient disposition were presented as means ± standard deviation and frequencies and percentages for the continuous and categorical variables, respectively. Patients were divided into two groups: survivors and non-survivors. In the bivariate analysis, Student’s t-test and Pearson’s chi-square test were used to compare the differences in the independent variables between both groups (continuous and categorical, respectively). Both tests were interpreted at a predetermined significance level (alpha = 0.05). A multivariate analysis using all statistically and clinically significant variables was performed using logistic regression to find the best model that fits the data and that explains the association between mortality and all predictor variables (including the L/A ratio). Variables included in the model were lactate/albumin ratio, Age, gender (reference: male), Chronic kidney disease, hypertension, dyslipidaemia, coronary artery disease, atrial fibrillation, malignancy history of stroke, history of Transient ischaemic attack (TIA), diabetes mellitus, chronic obstructive pulmonary disease, Systolic Blood Pressure (SBP) upon presentation, Heat rate (HR) upon presentation, O_2_ saturation upon presentation, respiratory rate upon presentation, q SOFA score, haemoglobin, platelets, bun, creatinine, bicarbonate, magnesium, calcium, phosphate, Vasopressor use in the first 24 h, patient received steroids, intubation within the first 24 h, intubation within the first 48 h. The magnitude of association between the predictor variables and mortality were determined by calculating the odds ratios (OR) and their corresponding 95% confidence intervals (CI) (Table 5). Receiver operating characteristic (ROC) curves were used to compare the accuracy of the L/A ratio and lactate in predicting mortality by obtaining their respective area under the curve (AUC). The ROC curve was used to determine the optimal cut-off of the L/A ratio (including sensitivity and specificity) that discriminates between survivors and non-survivors.

### Sample size calculation

Based on the retrospective study done by Bou Chebl et. al. at the same tertiary care centre, the AUC of lactate and L/A ratio were found to be 0.61 and 0.67, respectively. This indicated a difference of 0.06 performance units. Choosing a power of 80% and significance level of 0.05 a minimum sample size of 800 patients would be needed to detect a difference in AUC-ROC curves of 0.06 performance units. Because of ongoing recruitment for sepsis studies in our department between September 2018 and February 2021. We recruited 939 patients in order to further increase the power of the study.

## Results

### Characteristics of study subjects

**Figure 1.**
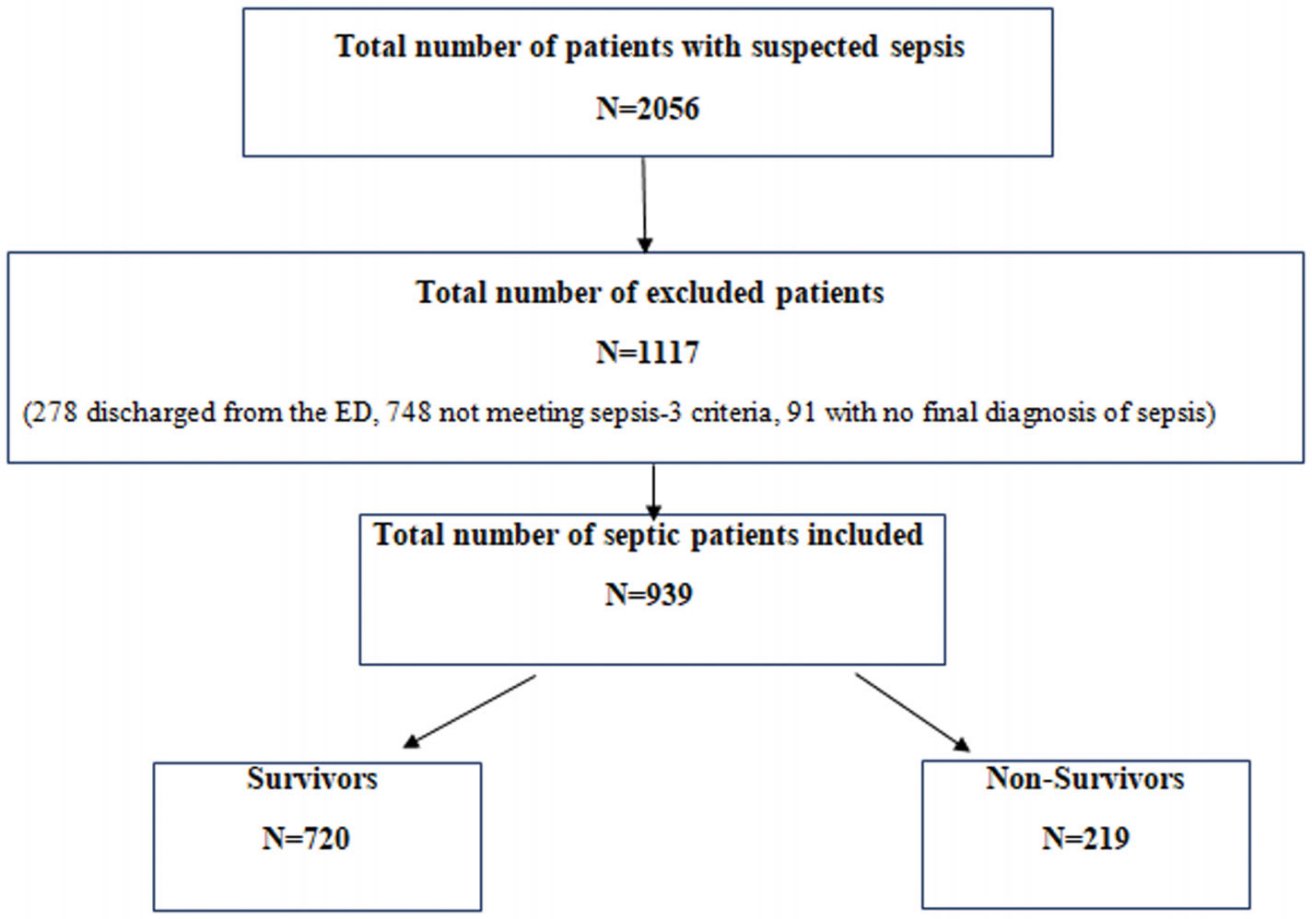
Flow diagram showing the included and excluded patients of the study. The process of patient recruitment based on the inclusion and exclusion criteria.

A total of 2056 patients with suspected sepsis were approached. A total of 939 septic patients were included throughout the study period (Figure 1). The average age of the included patients was 72.39 ± 15.62 years and 59.9% were males. 43.6% of the patients were smokers. The most common medical comorbidities were hypertension (63.6%), diabetes (40.3%), dyslipidaemia (40.1%) and current or a history of malignancy (39.6%). 23.3% (*N* = 219) of the patients presenting with sepsis died during their hospital stay (Table 1).

**Figure 2.**
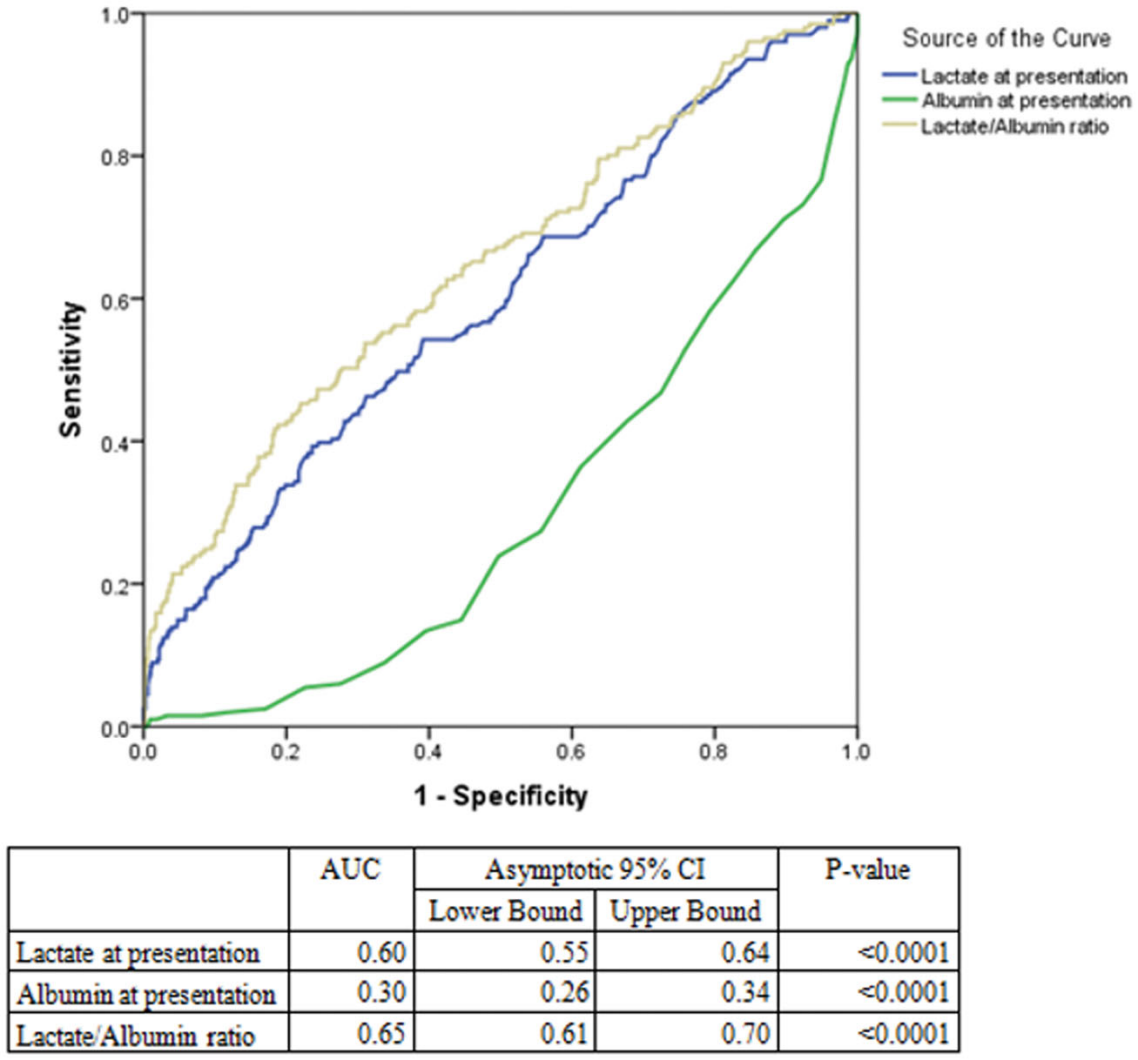
ROC curves for all septic patients (L/A ratio vs Lactate vs Albumin). Figure 2 shows the ROC aimed at comparing the AUC of lactate, albumin and lactate/albumin ratio among all septic patients in the study.

**Figure 3.**
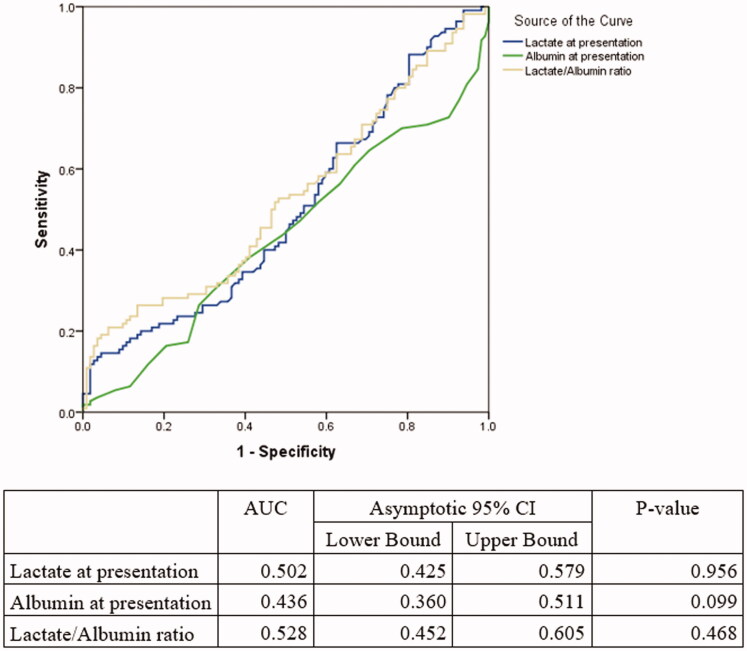
ROC curves for septic shock patients only (*N* = 236) (L/A ratio vs Lactate vs Albumin). Figure 3 shows the ROC aimed at comparing the AUC of lactate, albumin and lactate/albumin ratio among septic shock patients.

**Table 1.** Baseline characteristics of the patients presenting to the emergency department with sepsis or septic shock.

	Total (*N* = 939)	Survivors (*N* = 720)	Non-survivors (*N* = 219)	*P*-value
Age	72.39 ± 15.62	72.28 ± 15.88	72.74 ± 14.77	.71
Male	555 (59.1%)	400 (55.6 %)	155 (70.8%)	<.001
Smoking	409 (43.6%)	323 (44.9%)	86 (39.3%)	.14
Chronic kidney disease	220 (23.4%)	168 (23.3%)	52 (23.7%)	.9
Hypertension	597 (63.6%)	458 (63.6%)	139 (63.5%)	.97
Dyslipidemia	377 (40.1%)	293 (40.7%)	84 (38.4%)	.54
Atrial fibrillation	184 (19.6%)	143 (19.9%)	41 (18.7 %)	.71
Coronary Artery Disease	311 (33.1%)	237 (32.9%)	74 (33.8%)	.81
Congestive Heart Failure	229 (24.4%)	179 (24.9%)	50 (22.8%)	.54
Malignancy	372 (39.6%)	273 (37.9%)	99 (45.2%)	.05
History of stroke	72 (7.7%)	56 (7.8%)	16 (7.3%)	.82
History of Vascular Disease	88 (9.4%)	73 (10.1%)	15 (6.8%)	.14
Diabetes Mellitus	378 (40.3%)	286 (39.7%)	92 (42.0%)	.55
Chronic Obstructive Pulmonary Disease	155 (16.5%)	127 (17.7%)	28 (12.8%)	.09

Table 1 shows the patient characteristics and pre-hospital variables of septic/septic shock patients in order to compare survivors versus non-survivors.

### Survivors versus non-survivors

Several findings were significant between the survivor and non-survivor groups. The non-survivor group had a lower systolic blood pressure (114.74 mmHg vs. 121.23 mmHg *p* = .002), a higher heart rate and respiratory rate at presentation (103.32 bpm vs 99.29 bpm *p* = .04, 23.41 breaths/minute vs 21.07 breaths/minute; *p*-value < .0001). Moreover, the non-survivor group had a higher mean lactate level (3.78 ± 3.30 mmol/L vs 2.69 ± 1.70 mmol/L; *p* < .0001), a higher creatinine level (1.75 ± 1.22 mg/dL vs 1.49 ± 1.38 mg/dL; *p*-value = .02), and a lower mean albumin level (29.41 ± 6.84 g/L vs 34.14 ± 6.46 g/L with a *p* < .0001) (Table 2). Finally, the non-survivor group required more vasopressors and steroids in the first 24 h (31.5% vs 15.3%, 56.2% vs 23.2% respectively *p* < .0001 for both), as well as more intubation at 24 and 48 h (26% vs 6.4%, 17.4% vs 2.6% respectively with *p* < .0001 for both) (Table 3).

**Table 2.** Vital signs and laboratory parameters of patients presenting to the emergency department with sepsis or septic shock.

	Total (*N* = 939)	Survivors (*N* = 720)	Non-survivors (*N* = 219)	*P* value
Systolic blood pressure upon presentation (mmHg)	119.72 ± 26.61	121.23 ± 26.66	114.74 ± 25.87	.002
Diastolic blood pressure upon presentation (mmHg)	67.63 ± 15.65	67.92 ± 15.58	66.67 ± 15.87	.30
Heart rate upon presentation (Beats/minute)	100.23 ± 25.31	99.29 ± 25.28	103.32 ± 25.22	.04
Oxygen saturation upon presentation (%)	93.60 ± 9.55	95.15 ± 7.61	88.42 ± 12.97	<.0001
Temperature upon presentation (C)	37.43 ± 1.68	37.47 ± 1.73	37.30 ± 1.49	.21
Respiratory rate upon presentation (Breaths/minute)	21.61 ± 7.68	21.07 ± 7.38	23.41 ± 8.37	<.0001
Q sofa score > 2	221 (23.5%)	147 (20.4%)	74 (33.7%)	<.0001
White blood cell count (cu.mm)	11760.79 ± 9161.64	11631.76 ± 7472.00	12184.43 ± 13291.38	.56
Haemoglobin (g/dL)	11.40 ± 2.31	11.48 ± 2.26	11.13 ± 2.43	.05
Platelets (cu.mm)	223127.04 ± 135842.05	229452.25 ± 133595.17	202360.73 ± 141302.70	.01
Lactate (mmol/L)	2.94 ± 2.25	2.69 ± 1.70	3.78 ± 3.30	<.0001
CRP (mg/L)	132.79 ± 104.42	119.83 ± 101.45	161.70 ± 105.45	<.0001
Albumin (g/L)	33.06 ± 6.84	34.14 ± 6.46	29.41 ± 6.84	<.0001
Lactate/Albumin ratio	0.097 ± 0.091	0.083 ± 0.056	0.145 ± 0.140	<.0001
Procalcitonin (ng/mL)	5.7735 ± 16.79	5.44 ± 14.58	6.48 ± 20.76	.52
Glucose (mg/dL)	164.11 ± 91.32	165.77 ± 95.22	159.04 ± 78.23	.33
BUN (mg/dL)	33.71 ± 24.93	31.13 ± 22.99	42.19 ± 28.92	<.0001
Creatinine(mg/dL)	1.55 ± 1.35	1.49 ± 1.38	1.75 ± 1.22	.02
Bicarbonate (mmol/L)	23.72 ± 9.27	24.39 ± 9.75	21.60 ± 7.19	<.0001
Pao2	103.73 ± 66.66	105.68 ± 71.15	100.38 ± 58.19	<.0001
INR	1.59 ± 1.91	1.48 ± 1.77	1.84 ± 2.17	.05

Table 2 aims at comparing the initial vital and laboratory parameters upon presentation to the emergency department of both septic and septic shock patients between survivors and non-survivors.

**Table 3.** Therapeutic measures undergone and outcomes of patients presenting to the Emergency Department with sepsis or septic shock.

	Total (*N* = 939)	Survivors (*N* = 720)	Non-survivors (*N* = 219)	*P* value
Vasopressor use in the first 24 h	179 (19.1%)	110 (15.3%)	69 (31.5%)	<.0001
Patients who received steroids	290 (30.9%)	167 (23.2%)	123 (56.2%)	<.0001
Intubation within the first24hrs	103 (11.0%)	46 (6.4%)	57 (26%)	<.0001
Intubation within the first48hrs	57 (6.1%)	19 (2.6%)	38 (17.4%)	<.0001
Iv fluids infirst6hours	1.35 ± 1.04	1.37 ± 1.02	1.31 ± 1.10	.48
Iv fluids in the first_24_hours	2.19 ± 1.49	2.18 ± 1.48	2.22 ± 1.53	.70
Patients who developed septic shock	236(25.1%)	115(16%)	121(55.3%)	<.001
Patients admitted to the ICU	394(42%)	232(32.2%)	162(74%)	<.001
Patients who required mechanical ventilation	144(15.3%)	64(8.9%)	80(36.5%)	<.001
Average length of hospital stay	10.62 ± 12.70	8.93 ± 10.96	16.25 ± 16.09	<.001

Table 3 shows the therapeutic measures and associated outcomes of septic/septic shock patients among survivors versus non-survivors.

### Patient outcomes

Forty-two percent of the septic patients required intensive care unit admission and 15.3% required mechanical ventilation during their hospital stay. The percentage of patients that developed septic shock, required ICU admission and mechanical ventilation during their hospital stay was significantly higher in the non-survivor group (55.3% vs 16%, 74% vs 32.2%, 36.5% vs 8.9% respectively with a *p*-value < .0001 for all). The average length of hospital stay was significantly higher in the non-survivor group (16.25 ± 16.09 days vs 8.93 ± 10.96 days with a *p*-value < .001) (Table 3).

### Prognostic value of L/A ratio and lactate

The AUC value of the L/A ratio in septic patients was 0.65 (95% CI= [0.61 − 0.70]) and was higher than lactate alone 0.60 (95% CI= [0.55–0.64] with a *p* < .0001 (Table 4). The optimal L/A ratio cut-off threshold that separated survivors from non-survivors was found to be 0.115 for all septic patients (positive predictive value 39%, negative predictive value 83%, sensitivity 35%, specificity 81%) (Table 4, Figure 2).

**Table 4.** AUC and cut-off threshold of Lactate and Lactate to Albumin ratio within the different subgroups.

	AUC for in-hospital mortality (95% CI)			Lactate/albumin ratio cut-off threshold				
	Lactate	L/A ratio	*p*	Cut-off threshold	Sensitivity	Specificity	PPV	NPV
Overall	0.60 (0.55–0.64)	0.65 (0.60–0.69)	<.0001	0.115	0.35	0.81	0.39	0.83
Septic shock	0.50 (0.43–0.58)	0.53 (0.45–0.61)	.11	0.25	0.13	0.94	0.77	0.55
Lactate levels								
Lactate < 2 mmol/L	0.57 (0.50–0.64)	0.63 (0.55–0.70)	.07	0.035	0.89	0.29	0.219	0.924
Lactate ≥ 2 mmol/L	0.60 (0.54–0.66)	0.69 (0.64–0.74)	<.0001	0.12	0.61	0.68	0.39	0.84
Albumin levels								
Albumin < 30 g/L	0.66 (0.59–0.73)	0.69 (0.62–0.75)	.04	0.12	0.64	0.63	0.50	0.76
Albumin ≥ 30 g/L	0.52 (0.46–0.58)	0.55 (0.49–0.61)	<.0001	0.035	0.94	0.15	0.18	0.93
Patient subgroups								
chronic_kidney_disease	0.61 (0.51–0.70)	0.61 (0.52–0.71)	.66	0.125	0.32	0.88	0.44	0.82
Malignancy	0.62 (0.56–0.69)	0.68 (0.62–0.75)	<.0001	0.115	0.51	0.79	0.46	0.82
End Stage Liver Disease	0.74 (0.65–0.83)	0.77 (0.68–0.86)	.14	0.21	0.50	0.92	0.87	0.65
Infection site								
Lung	0.61 (0.56–0.66)	0.64 (0.59–0.69)	.001	0.07	0.66	0.54	0.40	0.77
Urine	0.69 (0.63–0.76)	0.76 (0.69–0.83)	<.0001	0.12	0.65	0.79	0.47	0.89
intravascular_catheter	0.84 (0.56–1.00)	0.87 (0.63–1.00)	.28	0.115	0.86	1.00	1.00	0.89
gastrointestinal	0.66 (0.51–0.81)	0.77 (0.63–0.91)	.001	0.135	0.65	0.86	0.52	0.91
Skin	0.42 (0.15–0.70)	0.58 (0.30–0.85)	.72	0.095	0.60	0.63	0.17	0.93
Heart	0.81 (0.68–0.94)	0.88 (0.77–0.98)	<.03	0.11	0.75	0.84	0.60	0.91
gall_bladder	0.56 (0.32–0.79)	0.65 (0.40–0.91)	.10	0.115	0.556	0.82	0.45	0.75
surgical_site	–	–	–	–	–	–	–	–
Bone	–	–	–	–	–	–	–	–
Peritoneum	0.61 (0.17–1.00)	0.68 (0.04–1.00)	.81	0.30	0.50	1.00	1.00	0.92
Diabetes	0.59 (0.51–0.66)	0.65 (0.57–0.72)	<.0001	0.115	0.49	0.78	0.43	0.83
Age								
<65	0.64 (0.55–0.72)	0.68 (0.59–0.76)	.001	0.095	0.56	0.74	0.37	0.86

Table 4 compares the prognostic usefulness of Lactate vs Lactate to Albumin ratio in predicting mortality among the different subgroups.

### Prognostic value of L/A ratio and lactate (subgroup analysis)

The AUC of the L/A ratio was significantly higher for patients with a lactate ≥2 mmol/L: 0.69 (95% CI 0.64–0.74) versus 0.60 (95% CI 0.54–0.66) with a *p* < .0001 (Table 4), as well as for patients with an albumin level less than 30 g/L (AUC = 0.69 95% CI= 0.62–0.75 vs AUC= 0.66 95% CI= 0.59–0.73, *p* = .04). In a similar manner, the ratio outperformed lactate alone in cancer patients (0.68 vs 0.62 *p*-value < .0001), in diabetic patients (AUC= 0.65 95% CI= 0.57–0.72 vs AUC = 0.59 95% CI= 0.51–0.66, *p* < .0001) as well as in patients older than 65 years of age (AUC= 0.63 95% CI= 0.58–0.69 vs AUC = 0.58 95% CI = 0.53–0.63, *p* < .001). Finally, when stratifying by infection source, the L/A ratio was a better prognostic marker than lactic acid alone in predicting mortality when sepsis was caused by respiratory, urinary and gastrointestinal (GI) infections (Table 4). Among septic shock patients, there was no statically significant difference in the AUC value of the L/A ratio compared to lactate (0.53 95% CI 0.45–0.61 vs 0.50 95% CI 0.43–0.58, respectively, with a *p*-value = .11) (Figure 3). In addition, among patients with end-stage liver disease, there was no statically significant difference in the AUC value of the L/A ratio compared to lactate (0.77 95% CI 0.68–0.86 vs 0.74 95% CI 0.65–0.83, respectively, with a *p*-value = .14). The optimal cut-off for the LA ratio in the ESLD group was 0.21 with a sensitivity and specificity of 50% and 92%, respectively (Table 4).

### Stepwise logistic regression for mortality

Lactate to albumin ratio was found to be associated with hospital mortality. For every 0.1 unit increase in the ratio, patients had 2.17 greater odds of mortality (OR = 2.17; 95% CI = [1.69–2.80] *p*-value < 0.0001). Patients who were intubated and who received steroids, also had greater odds of mortality (3.97 with a 95% CI = 1.84–8.54, *p*-value < .0001 and OR = 2.51 with a 95% CI = [1.65–3.82 *p*-value < .0001 respectively), whereas females had lower odds of mortality (OR = 0.61 with a 95% CI = [0.40–0.92] *p*-value = .018) (Table 5).

**Table 5.** Stepwise logistic regression for mortality as the primary outcome (including all septic patients).

	Mortality (reference: no)			
	OR	95% C.I.		*p* Value
		Lower	Upper	
Lactate/albuminratio	2.17	1.69	2.80	<.0001
Gender (female)	0.61	0.40	0.92	.018
Patientreceived steroids	2.51	1.65	3.82	<.0001
Intubation within first48hrs	3.97	1.84	8.54	<.0001

Variables included in the model.

Imposed: lactate/albumin ratio (increase by 0.1 unit).

Stepwise: Age; gender (reference: male); Chronic kidney disease; hypertension; dyslipidemia; coronaryarterydisease; atrialfibrillation; malignancy history of stroke; history of TIA; diabetesmellitus; chronicobstructivepulmonarydisease;SBP uponpresentation; HR upon presentation; O2 saturationuponpresentation; respiratoryrateuponpresentation;qsofa; haemoglobin; platelets; bun; creatinine; bicarbonate; magnesium; calcium; phosphate; Vasopressor use in the first 24 h; patient received steroids; intubation within the first24hrs; intubation within the first48hrs.

Table 5 shows the multivariate logistic regression showing the variable associated with higher mortality rates; a higher L/A ratio, female gender, use of steroids and those who were intubated within the first 48 h.

## Discussion

The results of this prospective study have shown that L/A ratio is a better prognostic marker than lactate alone in septic patients (AUC of L/A ratio 0.65, 95% CI= 0.61–0.70) versus lactate AUC= 0.60, 95% CI= 0.56–0.64) with a *p* <.0001. This superiority of the L/A ratio was also seen in several subgroups such as: lactate ≥2 mmol/L, albumin level less than 30 g/L, cancer patients, diabetic patients, patients older than 65 years of age and when stratifying by infection source. However, among septic shock patients, there was no statically significant difference in the AUC value of the L/A ratio compared to lactate alone. Furthermore, L/A ratio was found to be associated with in-hospital mortality (OR = 2.17; 95% CI = [1.69–2.80] *p*-value < .0001). Finally, the optimal L/A ratio cut-off threshold that separated survivors from non-survivors was found to be 0.115 for all septic patients.

It is well established in the literature that a single venous lactate value can be used as a reliable risk-stratification biomarker for patients who present to the ED with suspected sepsis and is an excellent prognostic biomarker for mortality and organ failure in the critically ill [4,9–11]. However, serum lactate is affected by many patient-related factors. Lactic acidosis/hyperlactataemia can be induced by commonly used medications such albuterol and metformin [5,12]. Liver disease can also impair lactate clearance causing increased blood levels [6]. Furthermore, some patients may be critically ill and still have a normal venous lactate, which could lead to false patient prognosis [13,14]. This can limit the reliable use of lactate individually in a high acuity setting like the Emergency Department [4,15].

Our results are in line with multiple studies looking at the importance of the L/A ratio in several conditions such as: sepsis (prospective study including 155 patients), heart failure (retrospective study including 4562 patients) and traumatic brain injury (retrospective study involving 273 patients). They found that the L/A ratio was a good predictor of mortality [16–18]. However, these studies were either retrospective in nature [16,18] or limited by their sample size [17]. The largest retrospective study was done by Gharipour et al. examined the role of the L/A ratio in 6000 septic patients and found that the ratio is significantly superior to a single lactate in predicting 28-day mortality (AUC: 0.69 vs 0.67 respectively) [19].

Albumin has been previously studied in sepsis and is even included in the APACHE II score commonly used to predict mortality in critically ill patients. Any hepatic dysfunction might affect its plasma level. It is also influenced by nutritional status and inflammation [14]. Given that several factors can influence both lactate and albumin levels, the L/A ratio can be used as a more reliable prognostic tool in septic patients.

An interesting finding in our data was that the L/A ratio was a better prognostic marker than lactic acid alone in predicting mortality when sepsis was caused by respiratory, urinary and GI infections, and this is consistent with a previous retrospective study we did [13]. One potential explanation could be those infections are the most common ones among elderly patients, a subgroup of population where the L/A ration outperforms lactate.

In our study, the AUC values of the L/A ratio and lactate in the septic shock subgroup were 0.53 and 0.50 respectively with no statistically significant difference between them. This is lower than what Wang *et al.* reported, where they found that the AUC of the L/A ratio in predicting mortality was 0.84 in septic shock patients [20]. This can be potentially explained by the low number of septic shock patients (*N* = 236). But more importantly, the lack of difference between both biomarkers could be due to the marked elevation of lactate in septic shock which would overcome albumin’s role.

The accuracy of lactate in predicting mortality in sepsis has been studied using different ranges. Trzeciak Et al. showed that a lactate ≥4 mmol/L had a sensitivity and specificity of 35% and 95% respectively in predicting early mortality (≤3 days) and 19% and 93% respectively in predicting in-hospital mortality [9]. In another study, a lactate ≥4 mmol/L had a sensitivity and specificity of 36% and 92% (with regards to in-hospital mortality) [4]. When a cut-off of 2.5 mmol/L or above was chosen sensitivity and specificity were found to be 59% and 71%, respectively) [4]. In our study, the optimal cut-off value of the L/A ratio that distinguishes survivors from non-survivors was 0.115 (which is equivalent to 1.15 due to the difference in the unit used for albumin in our study) with a sensitivity and specificity of 35% and 81% for all septic patients. This is slightly lower than Wang *et al.* (prospective study), Lichtenauer et al. (retrospective study), and Shin et al (retrospective study) who reported higher cut-off values at 1.32, 1.5 and 1.7, respectively [14,20,21]. Our cut-off was closer to Gharipour et al. (retrospective study) at around 1.01 (19). The optimal cut-off value is still a matter of debate and yet to be determined by future prospective studies.

It is interesting to note that when we stratified patients based on chronic medical conditions, the end stage liver disease subgroup had the highest LA ratio cut-off (0.21). This can be explained by the impaired liver clearance of lactate as well as the lower synthesis of albumin resulting in an increased LA ratio in this subgroup [6,14]. This subgroup also had the highest AUC (0.77) when stratifying patients based on medical comorbidities.

## Limitations

Our study has several limitations; it was conducted in a single tertiary-care centre that deals with complex and referral cases. We did not compare the AUC of the L/A ratio with validated scoring systems such as APACHE II in the ICU. Our study focussed on in-hospital mortality and did not include long term mortality after hospital discharge. Finally, the number of patients that developed septic shock was relatively low which may explain why there was no difference in the AUCs between L/A and lactate alone. A study involving a larger sample size with multiple centres would be the appropriate next step better evaluate the prognostic value of the L/A ratio and determine the optimal cut-off that discriminates between survivors and non-survivors.

## Conclusion

The L/A ratio proved to be a better predictor of in-patient mortality than lactate alone in sepsis patients. This pattern also applies across various subgroups in our study (malignancy, diabetics, age above 65, lactate level less than 2 mmol/L, albumin less than 30 g/L). This would provide ED healthcare providers with tools to risk stratify patients and predict hospital course, thus tailoring early management and interventions accordingly. However, they do not favour the use of the ratio over lactate in septic shock patients. Further studies should be done to evaluate the prognostic value of the ratio in patients with sepsis and septic shock.

## Data Availability

The data that support the findings of this study are available from the corresponding author [RBC], upon reasonable request. All the data were presented in the form of Tables. Patient information was collected from the electronic medical files of our institution.

## Prior meetings/presentations

The results/findings/material of this paper has not been publicly presented nor made available for public consumption.

## Abbreviations

L/A: Lactate/albumin
ED: Emergency Department
SOFA: Sequential organ failure assessment
SBP: Systolic Blood Pressure
HR: Heat rate
CBC: Complete Blood Count
BUN: Blood Urea Nitrogen
TIA: Transient ischaemic attack
OR: Odds ratios
CI: Confidence intervals
ROC: Receiver operating characteristic
AUC: Area under the curve
ICU: Intensive Care Unit
